# Pathology of the Mouth

**Published:** 1855-10

**Authors:** 


### ARTICLE VI.

### *Pathology of the Mouth.* By S. A. T.

WE have often been not a little surprised at the neglect which the diseases of the mouth have experienced at the hands of physicians. Dentists too are absorbed in the consideration of the malformations and disorders of the teeth, that they do not pay that attention to the mouth in general which the importance of its disturbances of function and alterations of structure demand. In the compass of an article so short as this is designed to be, we can do little more than call the attention of practitioners of dental surgery to certain points of interest connected with the pathology of the mouth, and leave them to prosecute the study for themselves. For physicians our remarks are not intended.

We would suppose that a consideration of the anatomical and physiological relations of the mouth to the rest of the economy would awaken sufficient interest in the mind of every one, to induce the closest attention to its morbid alterations. By means of its mucous membrane, it is connected by a continuous tissue with the lungs and the entire alimentary tract. Its copious vascular supply renders it directly dependent upon the heart, and causes it to reflect faithfully the various changes of strength and fulness which the organs of the circulation experience.

Its nervous connections are even more numerous and important. The great motor-nerves of the brain and upper part of the spinal cord nerve its muscles. The sensory filaments of the fifth pair confer upon it its delicate sensibility. A branch of that nerve and another of the glosso-pharyngeal enable the the tongue to appreciate the flavor of sapid substances. Thus, branches of the same nerve receive the impressions which induce its reflex movements.

It is thus brought directly in connection with that great nervous web which consists of the intricate interlacements of filaments from all these nerves, and which through the intervention of branches of the sympathetic becomes related to the entire system. A careful study of the relations of the plexiform ganglion, the tympanic plexus, the corda tympani, the otic ganglion, the spheno-palatine ganglion, the superior cervical ganglion, the cervical plexus and the various smaller interlacements of the sympathetic nerve along the vessels of the head will show abundant reason for that sympathy which exists between the mouth and every organ of the trunk and head.

The physiological links which bind the mouth to the rest of the body are also numerous and interesting. In the mouth we find the commencement of that series of processes which results in the nourishment of the entire frame. The sense of taste, which renders the necessary act of ingesting aliment a physical pleasure as well as a physical duty, resides here. Here, also, are performed these preliminary processes of insalivation, mastication, and commencing deglutition. Let the teeth be defective, and mastication consequently imperfectly performed, and a much greater burthen will be cast upon the stomach, for the same reason that a lump of alum will dissolve more slowly than a fine powder of the same salt.

So, too, if from any cause the saliva be not duly mingled with the food a positive arrest of digestion in certain articles of food will take place. It may be regarded as an established fact of physiology that the digestion of the farinaceous articles of food, or at least of the starch contained in them, is commenced in the mouth through the agency of the saliva. It has indeed been denied by Bernard and others, that this fluid acts otherwise than mechanically, viz. by separating, more minutely than mere trituration could do, the particles of the food.

Various arguments have been adduced in support of this position. Among others, it has been averred, that even admitting such a change to be capable of being produced by saliva, outside of the body, the exceedingly brief space of time in which it is subjected to this action in the mouth can be productive of no marked result, and that as soon as the mass arrives at the stomach, all action ceases. In regard to this simple fact, the greatest confusion prevails among observers. Some assert, that acids alone can prevent the metamorphosis of starch into sugar, by simply neutralizing the alkalinity of the saliva. In reply to this, however, it has been shown that strongly acid saliva still excited its peculiar influence upon this sort of food. Then it was said, that the gastric juice prevented the change, that if gastric juice were mixed with insalivated starch out of the body no sugar was formed, and especially was it insisted that this change did not take place within the stomach itself. Numerous experiments were made to determine the question. Fistulous openings were established in the stomachs of animals, and the changes of vessels, injected naturally and artificially, were observed, and the accounts given by different observers varied very remarkably. Some maintained what we have just said, others admitted that the change took place when gastric juice was mixed with the insalivated morsel out of the body, but not when the mixture was made in the stomach.

One cause of this discrepancy undoubtedly may be found in the manner in which the experiments were performed. In many instances the morsel was introduced directly through the fistulous opening into the mixed gastric juice and saliva in the stomach. Here, of course, no change could be looked for because the action could at most be superficial. Such experiments evidently could not be compared with those in which the morsel was masticated and insalivated in the ordinary way, and it was in these that the sugar was found in the contents of the stomach.

Some observers, however, failed to find sugar even when following the latter method. At first it was said, that this failure was because the sugar was absorbed as fast as it was formed. Such a reply as this, however, if accepted, would certainly make those chemists out to be wrong, who had found sugar in the contents of the stomach. Evidently, therefore, some other solution of the difficulty is to be sought.

[To be continued.]

